# The Domestication of Wild Boar Could Result in a Relaxed Selection for Maintaining Olfactory Capacity

**DOI:** 10.3390/life14081045

**Published:** 2024-08-22

**Authors:** Maria Buglione, Eleonora Rivieccio, Serena Aceto, Vincenzo Paturzo, Carla Biondi, Domenico Fulgione

**Affiliations:** 1 Department of Biology, University of Naples Federico II, 80126 Naples, Italy; maria.buglione@unina.it (M.B.); serena.aceto@unina.it (S.A.); vincenzo.paturzo@unina.it (V.P.); carla.biondi@unina.it (C.B.); 2 Department of Humanities Studies, University of Naples Federico II, 80133 Naples, Italy; eleonora.rivieccio@unina.it

**Keywords:** differential gene expression, olfaction, pig, phenotypic plasticity, RNA-seq, spandrel, *Sus scrofa*, transcriptome analysis, wild boar

## Abstract

Domesticated animals are artificially selected to exhibit desirable traits, however not all traits of domesticated animals are the result of deliberate selection. Loss of olfactory capacity in the domesticated pig (*Sus scrofa domesticus*) is one example. We used whole transcriptome analysis (RNA-Seq) to compare patterns of gene expression in the olfactory mucosa of the pig and two subspecies of wild boar (*Sus scrofa*), and investigate candidate genes that could be responsible for the loss of olfactory capacity. We identified hundreds of genes with reductions in transcript abundance in pig relative to wild boar as well as differences between the two subspecies of wild boar. These differences were detected mainly in genes involved in the formation and motility of villi, cilia and microtubules, functions associated with olfaction. In addition, differences were found in the abundances of transcripts of genes related to immune defenses, with the highest levels in continental wild boar subspecies. Overall, the loss of olfactory capacity in pigs appears to have been accompanied by reductions in the expression of candidate genes for olfaction. These changes could have resulted from unintentional selection for reduced olfactory capacity, relaxed selection for maintaining olfactory capacity, pleiotropic effects of genes under selection, or other non-selective processes. Our findings could be a cornerstone for future researches on wild boars, pigs, feral populations, and their evolutionary trajectories, aimed to provide tools to better calibrate species management as well as guidelines for breeders.

## 1. Introduction

Domestication is a complex phenomenon related to the wide variety of traits modified by artificial selection that has significantly transformed Earth’s biosphere and evolutionary trajectories of animals and plants [1,2,3].

During domestication, humans intentionally select some morphological, behavioural and physiological traits to enhance benefits and facilitate animal management (e.g., no cryptic coat color, reduction of fear and aggressiveness, increase of fatness) [4]. Other features, conversely, may often co-occur in domesticated animals as pleiotropically linked traits, such as: (i) reduction in brain size (especially in regions of the brain that control aggression and reactivity to external stimuli), (ii) delay in rates of development or paedomorphisis, (iii) changes in cranial morphology, (iv) retention of juvenile behaviours, (v) early sexual maturity, (vi) lop ears (thought to be the result of a failure of cartilage fully develop due to early sexual maturity) [5,6,7,8,9,10]. Another of these traits is the sense of smell which has a pivotal role for animals, enabling them to gather essential information about the surrounding environment and to engage in social communication [11,12].

Swine are considered to have one of the better-developed olfactory abilities, as suggested by the olfactory bulb of pigs that constitutes 7% of their brain size [13], and the porcine genome showing one of the largest olfactory receptor genes’ families [14,15,16,17], providing individuals the ability to perceive a large spectrum of odors. This is crucial for animals living in the wild, whose survival depends on their ability to perceive odours, i.e., to find food or water source [18], for the reproductive behaviour [19], for neonate–mother interactions [20] and to detect predators [21]. Moreover, the sense of smell in the wild boar is already active in an early stage of the gestation period, providing piglets with an advantageous pre-knowledge of the wild environment [22] which may potentially underlie the adaptability and invasiveness [23]. However, differences can often be found when comparing domesticated and wild animals, which are obviously exposed to different environmental stimuli [24,25,26,27]. For example, the density of primary sensory neurons in the olfactory mucosa, as well as the olfactory marker protein (OMP) and olfactory binding protein (OBP) expression level, is lower in pig than in wild boar [28,29], determining variations on the diet and on the microbiota [30]. 

However, although the sense of smell in porcine is widely studied, most of the analyses mainly focus on domestic forms and on aspects relevant for enhancing commercial pig production, as feed palatability and pheromones to stimulate reproduction [19,31,32,33], remaining several research questions unanswered. In particular, it is still poorly investigated: (i) what is the magnitude of the olfactory changes between the pig and the wild boar and (ii) which functional genes are involved in the differentiation of odorant perception. To address these questions, we set out to characterize gene expression pattern using a recent advance in transcriptome sequencing (RNA-seq). For the first time, we compared the transcriptome of the olfactory mucosa of domestic and wild forms of *Sus scrofa*, meaning pig and wild boar, respectively. In particular, we highlighted overall dissimilarities and identified variations in genes’ expression that could specifically correlate with olfactory differences in the two forms. Indeed, modifications in genes’ expression, although not affecting the structure of the encoded proteins, can alter abundance, timing or spatial distribution patterns of their products, providing a source of variation for phenotypic evolution [34,35,36,37].

The comparison was performed taking in mind the current and controversial taxonomic classification for the wild boar in different geographical subspecies [38,39,40]. Overlooking the taxonomic complexity of Italian wild boar, here we conventionally refer to continental wild boars as *Sus scrofa scrofa*, and to island wild boar as *Sus scrofa meridionalis* (from Sardinia). The latter appear to be descendants of ancient pig strains from an early stage of domestication, or nearly domesticated, escaped from humans control and readapted to the wild [41,42,43,44] in the early Neolithic [45,46], although actually it remains unclear whether the first swine repopulating the Sardinia were wild or domesticated [41]. 

*Sus scrofa scrofa*, instead, represents the wild boar forms widespread across Western Europe, more massive than its island counterpart and well adapted to Mediterranean scrub and forests [47,48,49,50,51].

## 2. Materials and Methods

### 2.1. Sampling Collection

We collected 3 Sardinian wild boars (40°5.21′ N/9°40.2′ E, Western part of the Ogliastra region, Sardinia, Italy) (2 females and 1 male), and 3 Southern Italian wild boars (40°30′ N/15°16′ E) (1 female and 2 males), aged ≥ 1 year, according to their dentation, coat color pattern and general aspect [52]. The wild boars were scarified during legal hunts in accordance with Italian National laws (157/92 and 394/91 Laws), and all field protocols were approved by the Ministry of Environment (ISPRA, protocol number 24581 10/07/2014). The good health of the animals was evaluated by a veterinary attending our sampling procedures.

In addition, we sampled 3 adult (2 females and 1 male) large white domestic pigs listed in the Indigenous Genetic Type Registry by the Italian National Association of Swine Breeders. 

Immediately after the animals’ death, we filleted ~7 cm section of tissue from the olfactory epithelium with a sterilized scalpel, placing the biological sample in RNAlater stabilization solution (Invitrogen) for immediate RNase inactivation. The samples were stored in laboratory at −20 °C until processing.

### 2.2. Transcriptional Profiling of Olfactory Mucosa

#### 2.2.1. RNA Isolation

Total RNA was isolated using RNeasy Mini Kit (Qiagen, Valencia, CA, USA) according to the manufacturer’s instructions. All the RNA samples were of high and comparable quality, as assessed by Agilent Bioanalyzer (Agilent Technologies, Boblingen, Germany). 

RNA library preparation and total RNA sequencing (2 × 100 paired-end) were generated according to standard procedures using Illumina Stranded Total RNA Prep with kit Ribo-Zero (Illumina Inc., San Diego, California, USA) on NovaSeq 6000 (Illumina, San Diego, CA, USA) at the Genomix4Life Srl (http://www.genomix4life.com/it/ accessed on 15 February 2024).

#### 2.2.2. Data Processing and Differential Expression Analysis

The initial raw sequencing reads were processed using Trimmomatic software v0.33 [53], trimming low-quality bases and removing any adapter sequences to ensure the quality and integrity of the data. 

The high quality reads obtained after trimming were then aligned to the *Sus scrofa* reference genome Sscrofa11.1 (available at https://www.ncbi.nlm.nih.gov/datasets/genome/GCF_000003025.6/ accessed on 20 May 2024) using the STAR (Spliced Transcript Alignment to a Reference) RNA-seq aligner [54]. STAR is known for its high accuracy and speed in mapping RNA-seq reads, and it handles spliced alignments efficiency.

Following the alignment, gene expression levels were quantified using RSEM (RNA-Seq by Expectation-Maximization) [55]. It constructs a gene expression matrix by estimating the number of RNA-seq fragments that originate from each gene, by modeling the sequencing process and accounting for factors such as transcript length and sequencing depth. The resultant expression matrix serves as the basis for differential expression analysis. 

The analysis of differentially expressed genes (DEGs) was performed using the edgeR software package v3.19 [56], designed for the analysis of RNA-seq count data and enable to employ statistical methods to identify genes that show significant changes in expression levels between different conditions. In this study, a stringent threshold was applied, with a False Discovery Rate (FDR) < 0.001 and Fold Change (FC) > 2, to ensure that the identified DEGs are both statistically significant and biologically relevant. 

To gain insights into the biological functions and processes associated with the differentially expressed genes, the Gene Ontology (GO) enrichment analysis was performed using ShinyGO (http://www.geneontology.org/ accessed on 25 May 2024) [57], which allows for the identification of GO terms that are significantly overrepresented among the DEGs, providing a functional context to the changes observed in the gene expression data.

## 3. Results

After quality filtering, 160,768,612 reads were successfully aligned to the Sus scrofa reference genome, with the percentage of mapped reads per sample ranged from 84.76% to 92.28% (Table 1).

**Figure 1:**
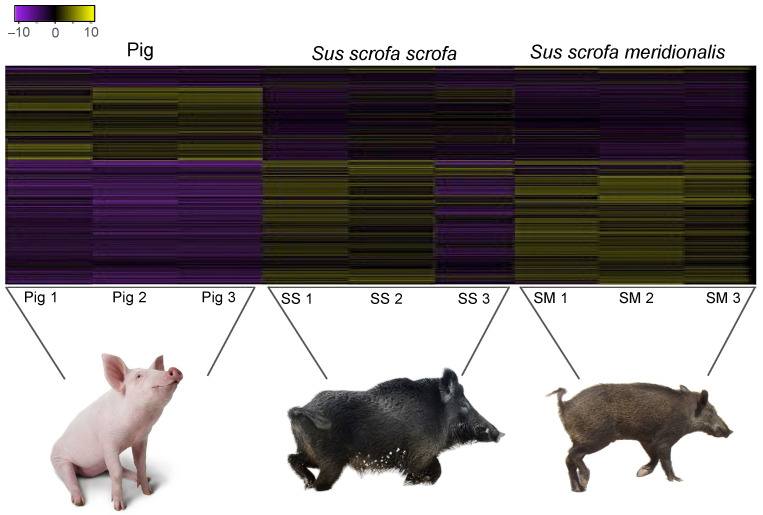
Heatmap of differentially expressed gene among the pig, Italian wild boar *Sus scrofa scrofa* (SS), and Sardinian wild boar *Sus scrofa meridionalis* (SM). The heatmap gives a graphical representation in expression level of the up (yellow) and down (purple) regulated genes.

The expression levels of transcripts in specific tissues were inferred through gene expression analysis visualized by a heatmap (Figure 1). This global expression pattern clearly delineates the differences between wild and domestic forms showing that numerous up-regulated genes in wild boar are either completely absent or down-regulated in pigs. A slight difference between the two wild boar’s subspecies was also observed (Figure 1).

The number of genes showing significant differential expression, with fold change (FC) of at least 2, was 918 between pig and *S. scrofa meridionalis* (with 603 up-regulated in *S. scrofa meridionalis*) and 384 between pig and *S. scrofa scrofa* (with 294 up-regulated in *S. scrofa scrofa*). In contrast, there are only 32 DEGs between the two wild boar’s subspecies (see Supplementary Material).

**Figure 2:**
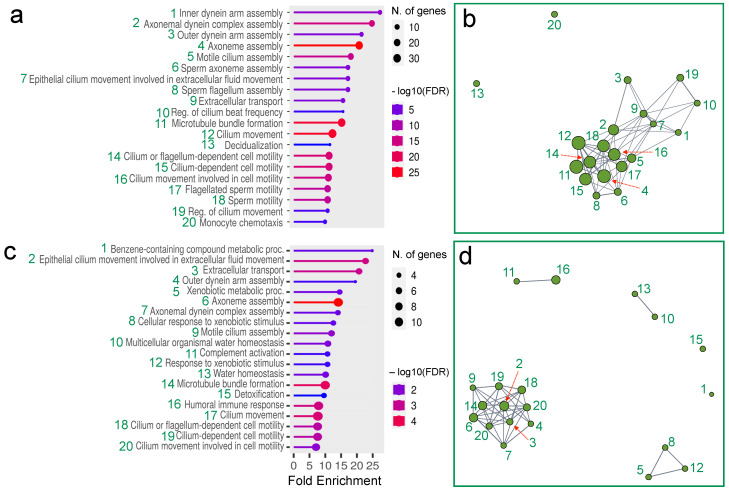
Gene Ontology enrichment analysis of the up-regulated genes in the olfactory mucosa of (**a**) Sardinian wild boar and (**c**) Italian wild boar, each compared to domestic pig. Linked-gene patterns (green spots) in (**b**) Sardinian wild boar and (**d**) Italian wild boar. The showed gene patterns are up-regulated in wild boar comparing to pig.

To better understand the potential functional relevance of the differences in gene expression, we examined the GO enrichment of the differentially expressed genes in wild boars compared to pigs (Figure 2), showing that in both Sardinian and Italian wild boars the most highly expressed genes are involved in the assembly of ciliary components and the regulation of ciliary movements. Interestingly, there is also an increased expression of genes typically associated with sperm motility and sperm flagellum assembly. 

Comparing pig with the two wild boars’ subspecies, we revealed differences in fold enrichment values (FCv): genes encoding motile cilium assembly exhibited nearly 15 FCv in Sardinian wild boar (Figure 2a) whereas in Italian wild boar the FCv value was estimated to be under 10 (Figure 2c). Similarly, genes associated with cilium or flagellum-depended cell motility, cilium-depended cell motility and cilium movement involved in cell motility showed more than 10 FCv in Sardinian wild boar (Figure 2a) and less than 10 FCv in Italian wild boar (Figure 2c).

Linked-gene pattern analysis for up-regulated genes in both in Sardinian wild boar (Figure 2b) and Italian wild boar (Figure 2d) demonstrates a close functional association of genes related to ciliary assembly and motility. Among them, genes up-regulated exclusively in Italian wild boar are involved in response to xenobiont, water and extracellular homeostasis, immune response, and benzene metabolism (Figure 2b,d).

**Figure 3:**
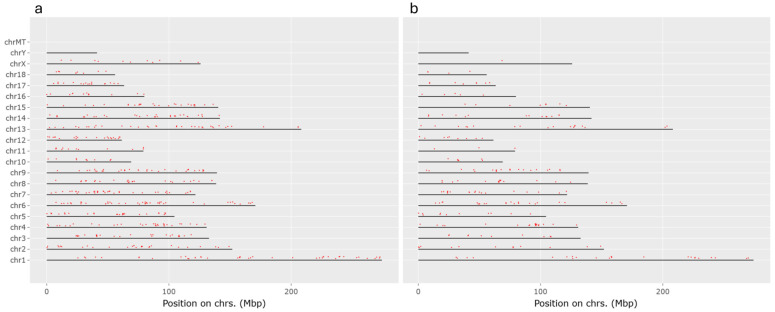
Gene position (red dot) on the chromosome (black horizontal line) in (**a**) Sardinian wild boar and (**b**) Italian wild boar. The genes are up-regulated in the wild boar compared to pig.

Analysis of the chromosomal locations of up-regulated genes in wild boars compared to pig reveals that the differences are primarily attributed to the greater number of up-regulated genes in Sardinian wild boars, particularly evident on chromosomes X, 18, 17, 15, 12, 11, and 7 (Figure 3).

## 4. Discussion

Artificial selection affects animals’ traits, shaping the phenotype in order to align with human-desired characteristics [1,58,59]. In particular, domestication of pigs has led to higher meat productivity, higher fertility and earlier sexual maturity, all aimed at enhancing food yield [25,43,60]. However, sometimes the captivity could lead to traits arising as byproducts, according to a spandrel effect [61]. For instance, while the sense of smell is crucial for survival in the wild, it may become less critical in controlled environments and does not directly impact on fitness of pigs. However, it is not clear if the loss of olfaction in the pig is the result of developmental constraints or without adaptive significance. Domestication could select for less sensitivity to odors, if for example it prevented unwanted behavioral responses to odors, such as the flight-or-fight response to natural enemies or intraspecific aggression. Conversely, domestication could result in relaxation of selection for maintaining olfactory capacity, which would be expected when the need to search for food is eliminated.

The reduction in odour perception of domestic *vs* wild swine forms has been studied by other authors using different approaches, i.e., with histological analysis of olfactory mucosa [28] or quantification of the expression of the single genes involved in sniffing [62]. We performed a comparative analysis of the olfactory mucosa of pig and wild boar by a whole transcriptome approach, never used before. We provided evidence of a significant reduction in the expression level of genes in domestic forms, supporting intriguing evolutionary implications, as the pleiotropy of traits emerging from domestication [9,63].

Our previous findings showed a greater quantity of mucosal cells and higher expression level of carrier proteins, as OBP and OMP, in wild boars [29,62], suggesting as the sense of small has crucial for survival and increasing fitness. Our present data enhance the understanding of this important toolkit for adaptation in wild. Indeed, we identify other candidate genes for reduction in olfactory capacity, such as those involved in the production of cilia, villi and microtubules, linked to catch odorous molecules and neurons’ functionality although they have other roles as well. Moreover, we hypothesize that down-regulated genes involved in the production and mobility of ciliary structures in pig, also influences genes typically associated with sperm motility and sperm flagellum assembly. This reflects the decreased activity in the production of microtubules, cilia and inner and outer dynein arms, involved in cell motility in the olfactory mucosa but linked to misleading GO assignment [64,65,66,67,68,69]. 

The down-regulation of genes linked to ciliary structures and sperm motility, although in a tissue not directly involved in the reproductive process, could suggest a lower reproductive performance in the domestic form which undergoes human manipulation to achieve more performing insemination. Indeed, domestication of animals appears to have had effects on the reproductive efficiency as suggested by ages of puberty and senescence, seasonality of breeding, sperm production [70]. In species managed by artificial insemination (AI), wild males showed higher sperm velocity, percentages of motile and progressive cells, and spermatocrit compared to domesticated individuals [71]. In domesticated pig AI affects the sperm performance in term of motility and dimension [72,73]. In Western Europe, more than 90% of the sows have been bred by AI for more than two decades [74,75], since that farmers consider AI a very useful tool to introduce superior genes into sow herds, with a minimal risk of disease [76]. Our data showed also the up-regulation of genes involved in immune response and benzene metabolism in Italian wild boars that may reflect adaptative strategies to respond to the wider variety of chemicals and pathogens in natural environments [77,78]. This would require a greater detoxification capacity and a more responsive immune system to protect the olfactory mucosa. Interestingly, the reduction of the expression of these genes is more pronounced when comparing pigs to Sardinian wild boars than to Italian wild boars. The differences in expression patterns between the two wild boar’s subspecies are confirmed and further highlighted by the chromosomal expression maps. We hypothesize that distinct phylogenetic histories influencing wild boar forms may contribute to this diversification. Specifically, ancestors of the Sardinian wild boar may have originated from ancient domesticated animals, introduced on island and abandoned or left to range freely thousand years ago [46,79]. The latter likely experienced more pronounced changes in the olfactory mucosa within just few generations compared to the adaptations observed in populations remained always wild which evolved to thrive in their natural environments. 

## 5. Conclusions

The sense of smell is crucial for macrosmatic animals living in the wild, like wild boar, whose survival depends on the ability to perceive odours. However, the artificial selection would have produced a reduction in the pig’s sense of smell probably because it is potentially redundant. For the first time, by using a whole transcriptome analysis, we performed a comparative investigation of gene expression in the olfactory mucosa of pig and wild boar, showing what is the magnitude of the olfactory changes between the domestic and wild forms and which functional genes are involved in the differentiation of odorant perception. Although the RNA-seq method is robust enough to not always require validation by other approaches [80,81,82,83,84,85], follow-up endeavors may be used to test these findings applying alternative quantification methods (i.e., qPCR) on a subset of genes to add value to our DEGs analyses.

Our empirical evidence could be a cornerstone for future researches on evolutionary trajectories of wild boars, pigs, and ferals allowing to address open issues that deserve to be developed. For instance, it would be interesting to extend the investigation to all domesticated breeds and wild boar’s subspecies differentiated globally. This huge survey, considering the different geographical locations, environmental conditions and farming methods, could clarify numerous aspects on the evolution of this intriguing species, providing tools to deal with wildlife emergencies as well as guidelines for breeders. For example understanding which genes can affect the adaptative abilities of wild populations is crucial to manage their invasive potential, mitigating or preventing their impact on biodiversity [23].

## Figures and Tables

**Table 1:** Statistics of the STAR mapping of the RNA-seq reads on the Sus scrofa reference genome. SS, Sus scrofa scrofa; SM, Sus scrofa meridionalis. s.d., standard deviation.

Sample	#Input Reads	%Mapped Reads	#Mapped Reads	#Mean Mapped Reads Per Type	s.d.
PIG_1	20,663,211	86.85	17,947,762	17,114,785	2,111,330
PIG_2	20,909,420	89.35	18,682,567		
PIG_3	17,359,635	84.76	14,714,027		
SS_1	26,605,201	86.36	22,976,252	19,000,282	3,580,300
SS_2	20,012,531	89.91	17,993,267		
SS_3	17,692,668	90.61	16,031,327		
SM_1	22,432,413	89.27	20,025,415	17,474,470	2,271,444
SM_2	17,523,002	89.43	15,670,821		
SM_3	18,126,543	92.28	16,727,174		

## Data Availability

All sequences data will be available in the Sequence Read Archive (SRA) in case of acceptance.

## Supplementary Materials

The following supporting information can be downloaded at: https://www.mdpi.com/article/10.3390/life14081045/s1, Table S1: Genes differentially expressed between pig and *Sus scrofa meridionalis* (SM), up-regulated in SM; Table S2: Genes differentially expressed between pig and *Sus scrofa meridionalis* (SM), up-regulated in pig; Table S3: Genes differentially expressed between pig and *Sus scrofa scrofa* (SS), up-regulated in SS. Table S4: Genes differentially expressed between pig and *Sus scrofa scrofa* (SS), up-regulated in pig.
